# Enhancing capacity for national genomics surveillance of antimicrobial resistance in public health laboratories in Kenya

**DOI:** 10.1099/mgen.0.001098

**Published:** 2023-08-30

**Authors:** Collins Kigen, Angela Muraya, Cecilia Kyanya, Leonard Kingwara, Onesmus Mmboyi, Tiffany Hamm, Lillian Musila

**Affiliations:** ^1^​ United States Army Medical Research Directorate-Africa, P. O. Box 606-00621, Village Market, Nairobi, Kenya; ^2^​ Jomo Kenyatta University of Agriculture and Technology, P. O. Box 62000-00200, Nairobi, Kenya; ^3^​ Wellcome Sanger Institute, Wellcome Genome Campus, Hinxton, Cambridge, CB10 1SA, UK; ^4^​ National Public Health Laboratory Services, Kenyatta National Hospital Grounds, Hospital Road, P.O Box 20750 -00202, Nairobi, Kenya; ^5^​ Henry M. Jackson Foundation for the Advancement of Military Medicine, 6720A Rockledge Drive, Bethesda, Maryland, USA

**Keywords:** antimicrobial resistance, genomics, WGS, public health, bioinformatics, Kenya

## Abstract

Genomic surveillance is vital for detecting outbreaks and understanding the epidemiology and transmission of bacterial strains, yet it is not integrated into many national antimicrobial resistance (AMR) surveillance programmes. Key factors are that few scientists in the public health sector are trained in bacterial genomics, and the diverse sequencing platforms and bioinformatic tools make it challenging to generate reproducible outputs. In Kenya, these gaps were addressed by training public health scientists to conduct genomic surveillance on isolates from the national AMR surveillance repository and produce harmonized reports. The 2-week training combined theory and laboratory and bioinformatic experiences with *Klebsiella pneumoniae* isolates from the surveillance repository. Whole-genome sequences generated on Illumina and Nanopore sequencers were analysed using publicly available bioinformatic tools, and a harmonized report was generated using the HAMRonization tool. Pre- and post-training tests and self-assessments were used to assess the effectiveness of the training. Thirteen scientists were trained and generated data on the *K. pneumoniae* isolates, summarizing the AMR genes present consistently with the reported phenotypes and identifying the plasmid replicons that could transmit antibiotic resistance. Ninety per cent of the participants demonstrated an overall improvement in their post-training test scores, with an average increase of 14 %. Critical challenges were experienced in delayed delivery of equipment and supplies, power fluctuations and internet connections that were inadequate for bioinformatic analysis. Despite this, the training built the knowledge and skills to implement bacterial genomic surveillance. More advanced and immersive training experiences and building supporting infrastructure would solidify these gains to produce tangible public health outcomes.

## Data Summary

The training exercise generated bacterial sequences for archived clinical multidrug-resistant *Klebsiella pneumoniae* from various healthcare facilities in Kenya on the most common sequencing platforms, Nanopore and Illumina. The sequences were deposited in the National Center for Biotechnology Information (NCBI) Sequence Reads Archive (SRA) public database under the accession numbers SRR22877381–SRR22877391 (BioProject accession PRJNA915250).

**Impact Statement**

This paper outlines the experience of conducting a genomics training programme in Kenya in a public health laboratory coordinating the national antimicrobial resistance (AMR) surveillance programme. We examine the challenges experienced when providing genomics training in low-resource settings, particularly within government institutions. We demonstrate the outputs of existing pipelines designed to streamline genomics surveillance analysis using bacterial isolates obtained through the surveillance programme. Finally, we make key recommendations for enhancing genomic surveillance of AMR in low-resource settings and offer guidance on implementing similar training programmes.

## Introduction

Antimicrobial resistance (AMR) is one of the most significant global threats to health, food safety and development [1]. The World Health Organization (WHO) has led the global effort to curb the rise in AMR by supporting the implementation of AMR national action plans. In Kenya, the AMR national action plan and surveillance strategies to track and prevent AMR were launched in 2017 and 2018, respectively [2, 3]. The national AMR surveillance programme, coordinated by the National Microbiology Reference Laboratory (NMRL) within the National Public Health Laboratories (NPHLs), currently includes 16 hospitals from over 13 counties in Kenya and the Kenya Medical Research Institute (KEMRI). These participating hospitals provide AMR susceptibility data and representative WHO target isolates to the national surveillance databases and the national isolate repository at the NMRL. The NMRL’s current role in national AMR surveillance is to offer reference testing, oversee quality assurance programmes in microbiology, train staff in the counties on how to conduct antimicrobial susceptibility tests (ASTs), provide standard operating procedures (SOPs), be a repository of bacterial isolates and publish national summary reports [4]. AST data from these hospitals were collated and submitted annually to the WHO’s Global Antimicrobial Resistance and Use Surveillance System (GLASS) [5] for 2021 and 2022. In addition, the NMRL currently has archived bacterial collections of reference strains from the AMR surveillance programme. One objective of the national surveillance strategy is to build the research capacity of the NMRL by archiving and analysing bacterial isolates using advanced techniques, such as next-generation sequencing (NGS), to determine the AMR genes, epidemiology and transmission of bacterial strains. The NPHL has significant whole-genome sequencing (WGS) capacity, with three sequencing technologies: Ion torrent, Illumina MiSeq and Oxford Nanopore, used predominantly for HIV, tuberculosis and coronavirus disease 2019 (COVID-19) analysis. Despite having this genomics capacity, the NMRL has not used it for AMR surveillance due to a lack of training and financial and human resourcing.

Efforts have been made across Africa to enhance capacity for WGS and bioinformatics in different fields, such as hereditary diseases and genomic diversity [6], to provide infrastructure for storage and analysis of genomic data (H3ABioNet) [7] and bioinformatics training [8, 9]. Initiatives such as SAGESA (https://github.com/WCSCourses/SAGESA-AMR-Genomics-Network), ACEGID (www.acegid.org) and WACCBIP (https://www.waccbip.org) have provided platforms to network and build capacity for genomic surveillance of AMR in Africa. Despite these and other investments, analyses of different genomic epidemiology studies conducted in sub-Saharan Africa still show that the region’s inadequate genomic research capacity hinders the full potential of genomics in medicine and research. They also highlight the importance of investing in the genomic and biotechnology sector, as doing so can lead to an increase in genomics research output. Therefore, it is crucial to focus on developing local capacity, particularly among individuals associated with universities in sub-Saharan Africa, where there are more opportunities for teaching and research [10]. To utilize genomics for bacterial epidemiology to address AMR, organizations such as the Public Health Alliance for Genomic Epidemiology (PHA4GE) (www.pha4ge.org) work with public health institutions to establish global standards and best practices around data and the availability of bioinformatic resources, and promote interoperability through tools such as hAMRonization (https://github.com/pha4ge/hAMRonization) and the credibility of public health bioinformatics. In addition, the Surveillance and Epidemiology of Drug-resistant Infections Consortium (SEDRIC) (https://sedric.org.uk/) promotes the power of data for public health use to tackle AMR and is working towards addressing some of these challenges globally and in low- and middle-income countries (LMICs).

Genomic epidemiology provides information on the geographical and hospital distribution of high-risk strains and detects outbreaks of highly pathogenic or multidrug-resistant (MDR) organisms to facilitate interventions to contain their spread. Genomic sequencing has recently become widely accessible and affordable, but its uptake in public health in LMICs such as Kenya is hampered by several challenges. First whole-genome sequences are generated using multiple platforms such as Illumina, Ion Torrent and Oxford Nanopore, all with different strengths, analysis parameters and pipelines. Second, WGS data for bacterial pathogens endemic to Kenya are typically generated by research and academic institutions such as KEMRI, but the sequences are not readily accessible to the national AMR surveillance platform, since the sequences are not always uploaded to public genome databases such as the National Center for Biotechnology Information (NCBI), the European Nucleotide Archive (ENA) (https://www.ebi.ac.uk) and the DNA Data Bank of Japan (DDBJ) (https://www.ddbj.nig.ac.jp). Third, although bioinformatics pipelines have been developed, e.g. ABRicate (https://github.com/tseemann/abricate), to analyse genomic data to detect AMR genes, these pipelines have varying output formats, and gene annotations make comparisons across pipelines very challenging. Finally, the interpretation of the output of these genomic pipelines into actionable data for public health interventions is a technical skill often lacking in public health laboratories.

Given the crucial role of genomic data for disease epidemiology and outbreak response, including informing rapid deployment of targeted interventions, there is a need to address gaps in capacity, infrastructure and sequence data sharing platforms between Kenyan country institutions playing a role in generating these data. For example, in KEMRI, AMR surveillance on WHO priority pathogens, including *Escherichia coli *, *Staphylococcus aureus *, *Klebsiella pneumoniae *, *Acinetobacter baumannii *, *Pseudomonas aeruginosa *, *Enterobacter* spp., *Salmonella* spp. and *Neisseria gonorrhoea*, among others, is ongoing in collaboration with various global partners. KEMRI uses its robust WGS Illumina and Nanopore platforms and bioinformatics infrastructure to examine the AMR genetic profiles and epidemiology of these bacterial pathogens [11–21]. This local expertise can be used to build similar capacity at the NMRL/NPHLs to benefit the national AMR surveillance programme. The goal would be to have a public health laboratory with the necessary number of staff trained in bioinformatics to generate and receive genomic data from other institutions, analyse them and generate outputs that benefit hospitals, public health response and policy. The funding received from PHA4GE in Kenya addressed some of the training, resourcing and genomic data interoperability and sharing gaps at the NMRL to build capacity in a public health institution to apply genomics to the surveillance of AMR and the establishment of a genomics surveillance network that supports national and global surveillance efforts. This report focuses on the training component of that funding effort, highlighting the successes and challenges experienced.

## Methods

### Training site

The training was held in Nairobi, Kenya, from 3–18 October 2022, at the NPHLs, where the lectures and wet and dry laboratory activities were conducted, and at the KEMRI Centre for Biotechnology Research and Development, where Illumina sequencing was performed.

### Isolates for the sequencing

Twenty-one anonymized multidrug-resistant *K. pneumoniae* isolates collected from patients within 2 prior years were retrieved from the NMRL repository for the training. These isolates were collected from nine hospitals in nine counties in Kenya. Only *K. pneumoniae* isolates were selected for the training to minimize variability in sample processing that could arise from working with different species. The selection criteria included MDR clinical isolates submitted to the National AMR surveillance repository. To ensure geographical diversity, isolates meeting the selection criteria were randomly selected from nine hospitals across nine counties in Kenya. The isolates were reidentified using matrix-associated laser desorption/ionization time-of-flight mass spectometry to confirm species identification. The NMRL also provided the AST results for each isolate. In addition, the sequences of two *K. pneumoniae* isolates, provided by the KEMRI AMR programme [15], with known AST and AMR gene profiles, were used as controls.

### Training manual

A training manual was created to guide the wet-laboratory processes, DNA extraction and quantification with the NanoPhotometer and Qubit fluorometer, and DNA library preparation and sequencing on Oxford Nanopore MinION and Illumina Miseq. The manual included worksheets to record experimental data and checklists for library preparation steps. Bioinformatic protocols were prepared as PowerPoint presentations and shared with the participants through a Google Drive folder.

### Training participants and trainers

A total of 13 trainees, 5 males, and 8 females, from different departments within NPHL laboratories (microbiology, genomics and molecular, and virology laboratories) and the KEMRI Center for Microbiology Research were selected to ensure a broad base of cross-disciplinary staff with bacterial genomics analysis skills. The selection criteria for participants in the training programme included being affiliated with one of two institutions, having a background in a genomics-related field or microbiology, and demonstrating interest in and availability to participate in the training. The trainers comprised one senior microbiologist and three molecular biology and bioinformatics scientists.

### Training approach

The training aimed to provide a combination of lectures on AMR genomics and hands-on experience in the laboratory bench procedures (wet laboratory) and bioinformatics analysis (dry laboratory). Etherpad (https://etherpad.org/), a web-based collaborative real-time editor, was used to share essential information, links, instructions and questions, allowing the participants to share their comments, feedback and responses. The lectures were conducted in an interactive session, with the trainers presenting slides and answering questions from the participants. For the wet laboratory, the participants were split into two groups of eight, each with a trainer. Both groups performed the same experiments using different *K. pneumoniae* isolates, each working on 10 isolates. The trainers first explained the concepts to the trainees before they performed the experiments, with the practical steps divided among each group member to ensure uniform participation. Each trainee recorded the experiment’s data in their worksheet in the training manual. The trainers covered the theory of bioinformatics analysis and demonstrated how to use the tools with an example dataset. The raw sequence reads generated in the wet laboratory were divided among the participants for analysis under the guidance of the trainers.

### Training modules

**Fig. 1.**
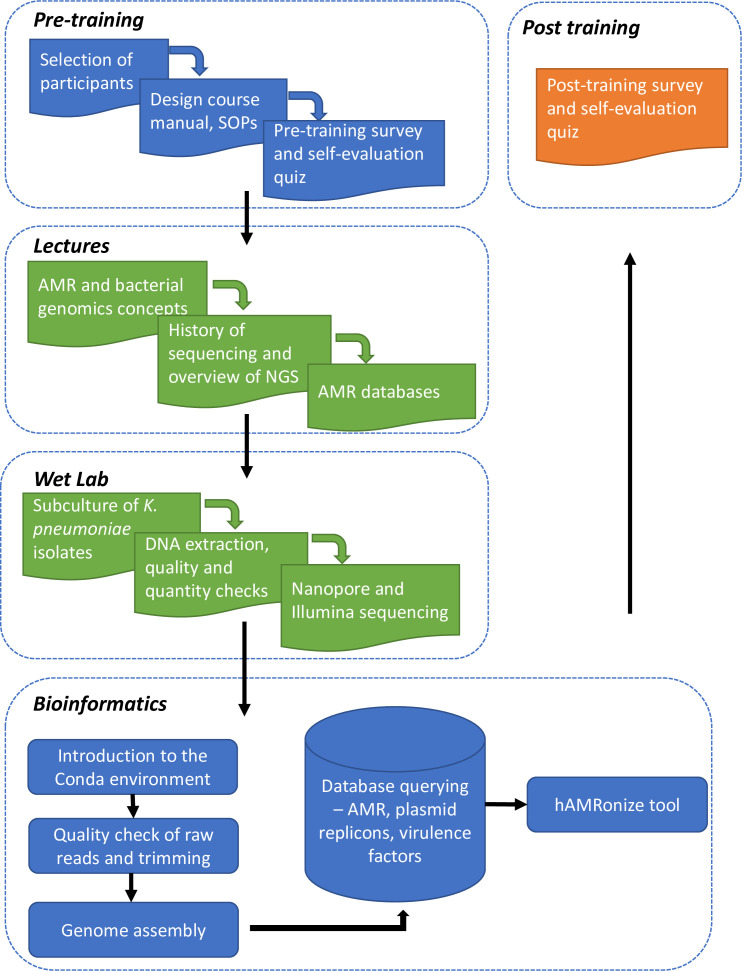
A flowchart illustrating the training process.

Training lectures covered key AMR and bacterial genomics concepts, the history of sequencing, and an overview of NGS technologies and the various AMR databases available. In the wet laboratory, the participants gained hands-on experience in DNA extraction, DNA quantification using Nanodrop and Qubit, and library preparation for Nanopore and Illumina sequencing. Finally, the training focused on bioinformatics, introducing the participants to the command line and Conda environment. Trainees learned about quality control checks of raw reads and genome assembly, database querying for AMR genes, plasmid replicons, virulence factors, and how to use the hAMRonize tool (https://github.com/pha4ge/hAMRonization) (Fig. 1)

### Evaluation of training impact

To evaluate the impact of the training on the trainees' knowledge and skills in AMR genomics, we administered both a pre- and a post-training survey based on self-evaluation and a pre- and a post-training quiz covering the topics discussed.

## Results and discussion

### Lectures and wet laboratory experience

The lectures were delivered in person and virtually, with good participant interaction and ample opportunity to clarify unfamiliar concepts. All participants had prior laboratory experience and were familiar with basic laboratory procedures and techniques. The combination of theory and practical activities solidified the learning experience. Retrieving isolates to analyse from the bacterial repository was challenging, as there was no electronic inventory, and the freezers were poorly organized. As a result, fewer isolates were retrieved than initially planned, and a non-* K. pneumoniae* isolate was unintentionally retrieved.

DNA extraction was initially performed using a Qiagen kit (QIAprep spin miniprep kit), which utilizes a chemical lysis method. This method was unsuccessful, as the DNA yield was less than the 1.5 µg needed as input for Oxford Nanopore sequencing. After troubleshooting, we determined that the kit was unsuitable for extracting DNA from *K. pneumoniae,* which is very mucoid and requires a more rigorous lysis method. We repeated the DNA extraction using a Zymo Research Quick Fungal/Bacterial Miniprep kit, which incorporates both mechanical and chemical lysis methods. The new kit yielded sufficient DNA (1.5–3 µg) for genome sequencing (Table S1, available in the online version of this article), which emphasizes the need to select suitable DNA extraction kits for the characteristics of the target bacterial species.

Before library preparation, DNA was quantified on a NanoDrop One spectrophotometer and a Qubit fluorometer and fragmented by repeated passage through a 1 mm syringe and needle. Then, library preparation for Nanopore was performed using the NEBNext companion module, the LSK109 ligation kit and the libraries loaded on R10.3 FLO-MIN111 flow cells. Collibri PCR-free ES kits and CD indexes were used for Illumina sequencing according to the manufacturer’s protocols.

### Dry lab

The bioinformatics training started with the installation of standard tools or analysis of whole-genome sequence data generated during the training. The participants used their own laptops, which required a Linux, Mac, or Windows operating system configured to support a Linux terminal. The Windows subsystem Linux was activated for the latter and the Ubuntu terminal v20.04.5 LTS was installed from the Microsoft store. The course first covered the fundamentals of the UNIX command line and the use of Conda as a package manager. Bioinformatic tools were installed using the Conda package manager before the bioinformatics training sessions and included the analysis tool hAMRonization (https://github.com/pha4ge/s). A high-performance computing server in KEMRI was used for analyses requiring high computational power, such as genome assembly of short reads using Shovill. Most trainees lacked prior command line experience. Therefore, the trainers took a personalized learning approach and used exercises to reinforce understanding for each participant to ensure they had mastered the concepts, as command line tools have a steep learning curve.

### Training challenges

The training faced several challenges, which could have affected its outcome. First, the absence of a dedicated project coordinator led to difficulties in managing course logistics, setting up laboratory supplies, and ensuring adequate computer and internet infrastructure. Second, working with government agencies and a central genomics laboratory with competing priorities also impacted on the project’s progress. For example, we had hoped to develop a repository for bacterial genomes using the generated sequences at the NPHL. However, this was delayed due to the ongoing migration of the server and the lack of established guidelines for granting NPHL servers access to other research institutions. A protocol for sharing sequence data to and from the genomic repository will be critical to facilitate sharing genomic data between institutions.

Supply chain challenges such as delayed deliveries and unavailability of preferred reagents added to the difficulties faced during the training. Internet connection issues also hampered the bioinformatics analysis. The larger number of participants than initially planned proved challenging, as it limited the hands-on engagement for all participants. The demand for such training was very high, and facilities and trainers were lacking to meet this demand. Some participants did not have laptops and had to share, limiting their experience. For other participants, the computing power of their laptops was limited and hindered analysis. Due to competing work engagements, as we were training in their duty stations, some participants could not fully participate in the training. In addition, several trainees were not full-time staff due to hiring freezes in the public sector. The inevitable staff turnover limits the sustainability of the long-term utility of the capacity built. Finally, frequent power outages interfered with the sequencing runs, leading to poor sequencing outcomes.

### Sequencing and genomic analysis results and reports

Sequencing of the 20 *K *. *pneumoniae* isolates on the Nanopore and Illumina platforms produced raw sequence data files for each isolate in FastQ format. In addition, the draft genomes of the isolates were used as inputs for screening for acquired AMR genes from AMR databases – CARD [22], ResFinder [23] and NCBI AMRFinderPlus [24] using the command-line-based ABRicate pipeline. ABricate was an ideal choice, as it produces multiple outputs from the different databases and is compatible with the hAMRonization tool. The outputs generated were in the form of tables in comma-separated versions. The tables were transformed and summarized using the hAMRonization tool into hAMRonized reports in an interactive HTML format.

From the AST results, high resistance was observed against the β-lactam antibiotics – cefuroxime (*n*=15), ceftriaxone (*n*=15), cefepime (*n*=15), ceftazidime (*n*=12), cefotaxime (*n*=11) and trimethoprim/sulfamethoxazole (*n*=12). The most common AMR genes conferred resistance to the beta-lactam (*bla*_CTX-M_, *bla*_OXA_, *bla*_TEM_), aminoglycoside (*aph*, *aac*), fluoroquinolone (*qnrB*, *qnrS*), trimethoprim (*dfrA*), sulphonamide (*sul1*, *sul2*) and tetracycline (*tetA*, *tetD*) antibiotic classes. There was a concurrence between the phenotypes and genotypes detected. The genomic analysis identified plasmid replicons in 17/20 isolates with the dominant plasmid from the IncF family. The IncF family is associated with the carriage of AMR genes and can be transmitted between *Klebsiella* and other *Enterobacterales* sp., accelerating the spread of AMR. The detailed outputs are reported in Table S2.

Participants acquired the skills to analyse, interpret and report on the resistance genes, correlate them with the existing AST data in these pathogens, and identify plasmids that can transmit these genes between and within species. These genomic data would be more robust when combined with the existing metadata on the hospitals, counties, specimen types and clinical profiles of the patients, which exceeded the scope of the training. The output indicates the potential to conduct in-depth analysis on hospital outbreaks and transmission within and between counties and AMR trends when data are generated for a more extensive isolate pool.

### Training impact

We assessed the impact of the training by conducting pre- and post-training tests and having the participants assess their competence levels. We compared the percentage of participants that scored 3–5 for competence on a scale of 1–5, with 5 being the highest score, before and after the training for different aspects. The percentage of participants scoring 3–5 in bacterial genomics concepts improved from 20–72.7 %, in antimicrobial resistance theory from 60–72.7 %, in sequencing and NGS technologies from 41.2–81.8 %, in Illumina sequencing from 36.4–81.8 %, in Nanopore sequencing from 30.8–81.8% and in the use of command line interface from 0–63.6 %. The self-reported competence gains were mirrored in the pre- and post-test results. For example, of the 10 trainees who took the test, 9/10 increased their scores, with an average increase of 14 % (32–46 %). There was an overall increase in self-assessed competence across all training areas, ranging from ~2- to 4-fold. The greatest gains in competence were in bacterial genomics, Nanopore and Illumina sequencing, and using the command line interface. ((Fig. S1) To ensure sustainability in bioinformatics analysis, we donated a new laptop to the NPHL genomics department equipped with a Linux operating system, 16 GB RAM, a core i7 CPU running @2.90 Hz with 12 cores and 512 GB SSD storage capacity.

### Recommendations

Our training experience in a limited resource setting highlighted a few valuable lessons for genomics and bioinformatics training in similar settings. Grouping the trainees according to their competence levels and maintaining a 1 : 5 trainer: trainee ratio is ideal to ensure that trainees learn at their own pace. To enhance continued participant engagement, exempting participants from regular work duties during training would make it a more productive learning experience. We also recommend that each participant has a laptop or Personal Computer with at least 8 GB RAM and eight cores. In instances where the physical computing infrastructure is lacking or unaffordable, cloud computing platforms such as Terra, which allow for data hosting, analyses and sharing, are better alternatives. In addition, a fast and stable internet with enough bandwidth should be provided to support bioinformatics analysis. Backup power should be provided for the critical sequencing runs and bioinformatic sessions to mitigate power outages. Logistics could be streamlined by ensuring that procurement for equipment, training materials and consumables is done well in advance to avoid disruption of the training due to delays and by having a designated training coordinator handle all supplies and daily training set-ups for the lecture and laboratory sessions. Given the complexity of bacterial genomics, short-term training like this is suitable for building foundational skills in genomics. However, a longer-term immersive experience with experienced scientists is necessary for a more significant impact and would produce trainers of trainers to mitigate staff turnover. We recommend a practical assessment in which trainees recreate the analysis without support from the trainers as a more objective assessment of bioinformatic competence. Finally, a comprehensive evaluation of public health laboratories concerning implementation of genomics using the Pathogen Genomics in Public Health Surveillance Evaluation (PG-PHASE) framework is recommended [25] to assess long-term sustainability and gains.

## Conclusion

The introduction of genomic epidemiology to public health laboratories in LMICs provides a necessary bridge to identifying and averting bacterial threats within hospitals. It provides critical evidence to implement infection and prevention control measures and other strategic interventions to avoid public health emergencies. Although sequencing capacity now exists in Kenya, critical gaps in staff education on new and robust sequencing technology and bioinformatics still exist. Some of this capacity resides in temporary staff or trainees who do not contribute to a stable base of expertise in the public sector. Funding for genomics and bioinformatics often vastly underestimates the resource gaps in LMICs, which range from engaged and permanent staff, freezer inventory systems for bacterial isolates, adequate cold storage for reagents, benchtop tools such as pipettes, reliable suppliers of reagents such as molecular-grade water, sequencing kits, adequate server storage, and reliable and stable power and internet bandwidth. Funding levels for such projects should be adequate to cover such gaps. While pipelines such as those designed by PHA4GE have provided a promising solution to the critical challenge of coherent and harmonized bioinformatic analysis, these overlooked gaps will hinder the best efforts of funders and partners. Since the greatest burden of AMR in the future is predicted to be borne in sub-Saharan Africa, this initial effort to incorporate genomic epidemiology into the national AMR surveillance system has provided a foundation on which to increase the understanding of the scale, distribution and impact of AMR genes and bacterial strains in Kenya and respond with the required urgency.

## Supplementary Data

Supplementary material 1
