# Supplementary material for: Enhancing capacity for national genomics surveillance of antimicrobial resistance in public health laboratories in Kenya
Source: Microb Genom. 2023 Aug 30;9(8):mgen001098. doi: 10.1099/mgen.0.001098 (PMC10483422; doi:10.1099/mgen.0.001098)
Supplement: Supplementary material 1 [file mgen-9-1098-s001.pdf]

Fig. S1: The figure represents the level of competency in the course topics before (blue) and after (orange) the training, based on the self-assessments conducted by the participants. The x-axis represents different levels of competency (1 to 5) with 1 being the lowest and 5 being the highest, while the y-axis shows number of participants who rated themselves each level. The graph highlights the significant improvement in participants' self-assessment level of competency in all course topics after the training program.

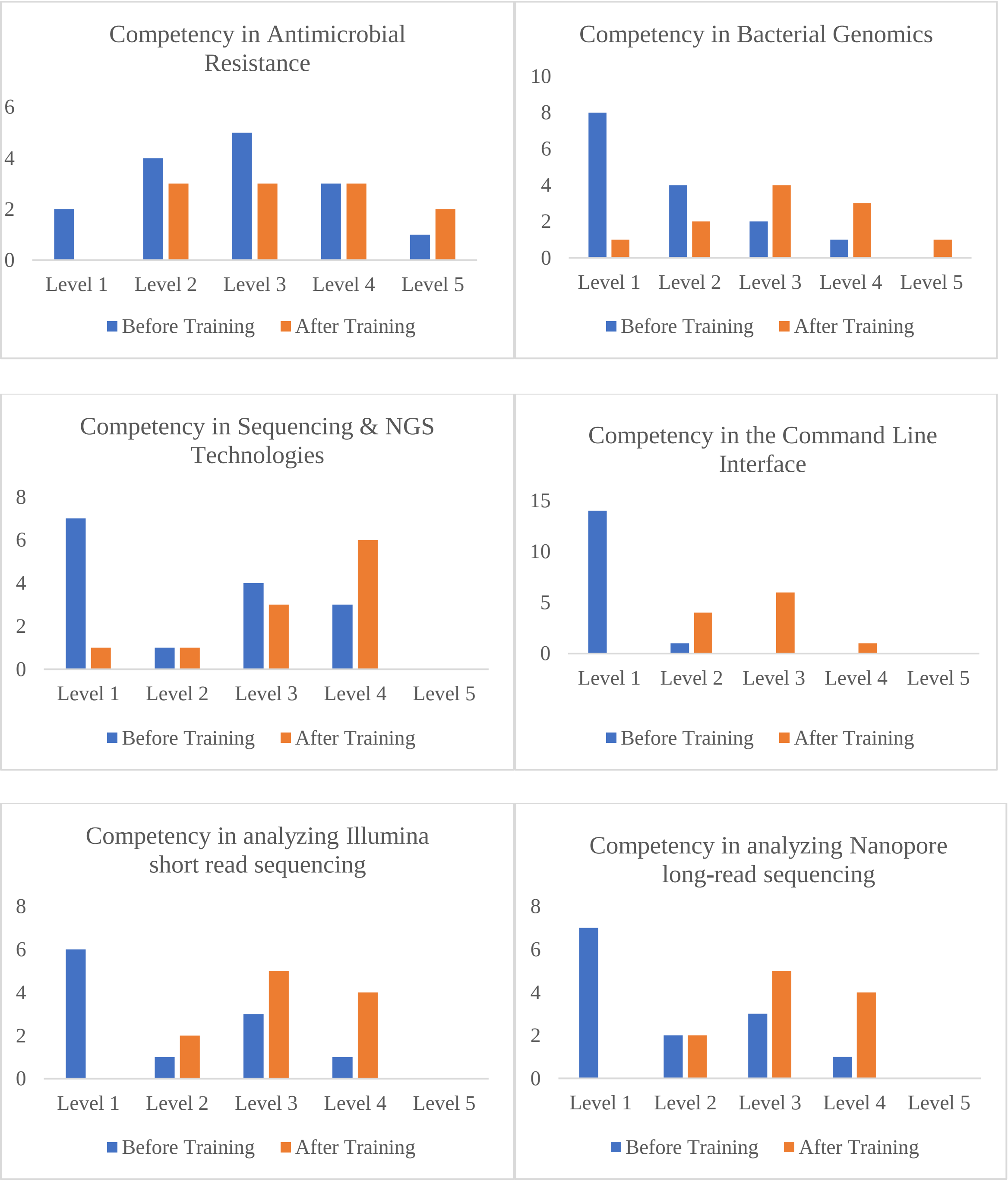

Table S1: DNA concentration, A260/280 ratios, A260/A230 ratios from NanoDrop One assays, and concentration from Qubit assay of the 21 *K. pneumoniae* isolates used for the training

**Sample number**	**Nanodrop One Assay**			**Qubit Assay**
	**Concentration (ng/µl)**	**A260/A280**	**A260/A230**	**Concentration (ng/µl)**
1A	167.6	1.923	1.665	89.5
1B	164.8	1.91	0.317	86.7
2A	92.2	1.826	0.188	80.2
2B	131.7	1.873	1.743	81.8
3A	101.5	1.938	1.087	83.3
3B	139.15	1.905	1.564	81.4
4A	171.65	1.848	0.104	85.4
4B	172.55	1.893	1.768	83.9
5A	151.25	1.906	0.197	88.5
5B	146.6	1.882	1.477	85.8
6A	199.6	1.897	1.712	89.9
6B	138.4	1.629	0.064	88.51
7A	102.55	1.687	0.059	79.7
7B	108.35	1.697	0.112	82.5
8A	162.7	1.921	1.474	87
8B	168.05	1.922	1.316	86.4
9A	45.7	1.41	0.594	42.6
9B	66.35	1.937	0.96	66.2
10A	51.95	1.729	0.048	41.6
10B	72.1	1.832	0.65	69
11A	263.7	1.912	1.405	91

Table S2: Outcome of analysis of AMR genes using the hAMRonize tool in phenotypically characterized *K. pneumonia* isolates from the Kenya national AMR Surveillance and plasmid replicons identified.

Isolate	County	Specimen type	Phenotypic Resistance	Plasmid Replicons	AMR genes	Class of antibiotic - genotypic resistance
Kp01	Kiambu	Blood	Amoxicillin/clavulanic, ampicillin/sulbactam, cefazolin, cefuroxime, cefuroxime axetil, cefotaxime, ceftazidime, ceftriaxone, cefepime, aztreonam, gentamicin, nitrofurantoin, piperacillin/ tazobactam, trimethoprim/sulfamethoxazole	IncFIB(K)_1_Kpn3, IncFII_1_pKP91	*AAC (3)-Iie, APH(3'')-Ib, APH(3')-Ia, APH(6)-Id, CTX-M-114, SHV-11, TEM-1, TEM-215, acrB, aph(3'')-Ib, blaCTX-M-114, blaSHV-11, blaTEM-1, blaTEM-215, dfrA14, emrR, mph(A), sul2*	Aminoglycoside, Beta-lactams, Diaminopyrimidine, Macrolides, Sulphonamides
Kp02	Kiambu	Urine	cefuroxime, cefotaxime, ceftazidime, ceftriaxone, cefepime, aztreonam, ciprofloxacin, piperacillin/ tazobactam, trimethoprim/sulfamethoxazole	IncFIB(K)_1_Kpn3, IncFII_1_pKP91	*AAC(6')-Ib-cr, APH(3'')-Ib, APH(3')-Ia, APH(6)-Id, CRP, CTX-M-15, FosA5, H-NS, OXA-1, QnrB17, SHV-106, TEM-1, TEM-166, aac(6')-Ib-cr10, acrB, acrD, baeR, blaCTX-M-188, blaOXA-1, blaTEM-166, cpxA, dfrA14, emrR, marA, mdtB, mdtC, msbA, oqxA, oqxB, ramA, sul2*	Aminoglycosides, Beta-lactams, Fosfomycin, Fluoroquinolones, Sulphonamides, Diaminopyrimidine,
Kp03	Bungoma	Blood	cefuroxime, ceftriaxone, cefepime, ciprofloxacin, trimethoprim/sulfamethoxazole	IncFIB(K)_1_Kpn3, IncN_1	*ADC-73, APH(3'')-Ib, APH(6)-Id, CTX-M-15, OXA-23, OXA-66, abeABC , armA, dfrA14, mph(E), msr(E), sul2, tet(B), tet(C)*	Aminoglycoside, Beta-lactams, Fluoroquinolone, Macrolide, Phenicols, Rifamycins,

						Sulfonamide, Tetracycline, Diaminopyrimidine
Kp04	Nakuru	Blood	cefuroxime, ceftazidime, ceftriaxone, cefepime, ciprofloxacin	IncFIB(K)_1_Kpn3, ColRNAI_1	*APH(3'')-Ib, APH(6)-Id, CRP, FosA6, H-NS, SHV-11, acrB, acrD, baeR, cpxA, dfrA5, emrR, marA, mdtB, mdtC, msbA, oqxA, oqxB, ramA, sul2, tet(D)*	Aminocoumarin, Aminoglycoside, Beta-lactams, Fluoroquinolone, Fosfomycin, Macrolide, Fluoroquinolone, Rifamycin, Sulfonamide, Tetracycline, Diaminopyrimidine
Kp05	Nyeri	Pus swab	ceftriaxone, cefepime, meropenem, gentamicin, tigecycline, piperacillin/ tazobactam, trimethoprim/sulfamethoxazole	IncX3_1, IncFIB(K)_1_Kpn3, IncFII_1_pKP91, IncFIA(HI1)_1_HI1	*AAC(6')-Ib-cr, APH(3')-Ia, CRP, CTX-M-15, FosA6, OXA-1, QnrS1, SHV-134, TEM-1, aadA2, acrB, acrD, baeR, cpxA, dfrA12, dfrA15, emrR, mdtB, mdtC, msbA, oqxA, oqxB, sul1, tet(A),*	Aminoglycoside, Fosfomycin, Beta-lactams/inhibitors, Fluoroquinolones, Sulphonamides, Tetracyclins, Aminocoumarin, Diaminopyrimidine, Fluoroquinolone, Nitrofuran, Nitroimidazole, Triclosan,
Kp06	Kilifi	Urine	nitrofurantoin	No replicons detected	*CRP, FosA5, H-NS, SHV-187, acrB, acrD, baeR, cpxA, emrR,*	Aminocoumarin, Aminoglycoside, Beta-lactams,

					marA, mdtB, mdtC, msbA, oqxA, oqxB, ramA	Diaminopyrimidine, Fluoroquinolone, Fosfomycin, Macrolide, Fluoroquinolone, Tetracycline, Triclosan
Kp07	Kiambu	Urine	amoxicillin/clavulanic, ampicillin sulbactam, cefazolin, cefuroxime, cefuroxime axetil, cefotaxime, ceftazidime, ceftriaxone, cefepime, aztreonam, gentamicin	IncFII_1_pKP91, IncFIB(K)_1_Kpn3, Col440I_1	*AAC(3)-IId, APH(3'')-Ib, APH(3')-Ia, APH(6)-Id, CRP, CTX-M-15, FosA6, H-NS, SHV-187, TEM-1, TEM-106, acrB, acrD, aph(3'')-Ib, aph(3')-Ia, aph(6)-Id, baeR, blaTEM-1, cpxA, dfrA14, emrR, marA, mdtB, mdtC, mphA, msbA, oqxA, oqxB, ramA, sul2,*	Aminocoumarin, Aminoglycoside, Beta-lactam, Diaminopyrimidine, Fluoroquinolone, Fosfomycin, Glycylcycline, Macrolide, Nitrofuran, Nitroimidazole, Phenicol, Rifamycin, Sulfonamide, Tetracycline, Triclosan
Kp08	Kisumu	Stool	cefazolin, cefuroxime, ceftazidime, ceftriaxone, cefepime, meropenem, gentamicin, ciprofloxacin, nitrofurantoin, trimethoprim/sulfamethoxazole, ertapenem	No replicons detected	*Klebsiella_pneumoniae_KpnG*<br><br>(NB: Sequence was incomplete)	Aminoglycoside, Carbapenem, Cephalosporin, Fluoroquinolone, Macrolide, Peptide

Kp09	Nyeri	Pus	ampicillin/sulbactam, cefazolin, cefuroxime, cefuroxime axetil, cefotaxime, ceftazidime, ceftriaxone, cefepime, aztreonam, gentamicin, trimethoprim/sulfamethoxazole	IncFIB(Mar)_1_pNDM-Mar, IncFII(pRSB107)_1_pRSB107, IncFIA(HI1)_1_HI1, IncFIB(K)_1_Kpn3, IncHI1B_1_pNDM-MAR, ColRNAI_1	*AAC(3)-IIe*, *AAC(6')-Ib-cr*, *APH(3'')-Ib*, *APH(6)-Id*, *CRP*, *CTX-M-15*, *OXA-1*, *QnrS1*, *TEM-1*, *aadA5*, *dfrA17*, *oqxA*, *oqxA10*, *sul1*, *sul2*, *tet(A)*,	Aminoglycoside, Beta-lactam, Diaminopyrimidine, Doxycycline, Fluoroquinolone, Glycylcycline, Macrolide, Nitrofuran, Phenicol, Sulfonamide, Tetracycline
Kp10	Kisumu	Urine	ampicillin/sulbactam, cefazolin, cefuroxime, cefuroxime axetil, cefotaxime, ceftazidime, ceftriaxone, cefepime, aztreonam, gentamicin, nitrofurantoin, trimethoprim/sulfamethoxazole	IncFII_1_pKP91, IncFIB(Mar)_1_pNDM-Mar, IncFIB(K)_1_Kpn3, IncHI1B_1_pNDM-MAR	*AAC(3)-IIe*, *AAC(6')-Ib-cr*, *APH(3'')-Ib*, *APH(6)-Id*, *CRP*, *CTX-M-15*, *FosA6*, *FosA7*, *H-NS*, *OXA-1*, *QnrB17*, *TEM-1*, *acrB*, *baeR*, *cpxA*, *dfrA14*, *emrR*, *mdtB*, *mdtC*, *msbA*, *oqxA*, *oqxB*, *sul2*, *tet(A)*	Aminocoumarin, Aminoglycoside, Beta-lactams, Diaminopyrimidine, Fosfomycin, Macrolide, Fluoroquinolone, Nitrofuran, Nitroimidazole, Phenicol, Rifamycin, Sulfonamide, Tetracycline, Triclosan
Kp12	Muranga	Sputum	ampicillin/sulbactam, cefazolin, cefuroxime, cefuroxime axetil, cefotaxime, ceftazidime, ceftriaxone, cefepime,	IncI1_1_Alpha, IncFIB(K)_1_Kpn3, IncL/M(pOXA-48)_1_pOXA-48,	*ANT(3'')-IIa*, *APH(3'')-Ib*, *APH(6)-Id*, *CRP*, *CTX-M-15*, *H-NS*, *QnrS1*, *SHV-40*, *TEM-1*, *aadA1*, *aadA2*, *acrB*, *acrD*, *aph(3'')-Ib*, *aph(6)-Id*, *blaCTX-M-*	Aminocoumarin, Aminoglycoside, Macrolide, Beta-lactam, Phenicol, Diaminopyrimidine,

			aztreonam, trimethoprim/sulfamethoxazole	IncFIB(K)_1_Kpn3, IncFII(pCRY)_1_pCRY, ColRNAI_1	*15, blaSHV-209, catI, cmlA1, cpxA, dfrA12, dfrA7, emrR, mdtB, mdtC, mph(A), oqxA, oqxB, qacH, qnrS1, sul1, sul2, sul3, tet(A)*	Tetracycline, Fluoroquinolone, Nitrofuran, Rifamycin, Sulfonamide, Triclosan
Kp13	Machakos	Aspirate	No phenotypic resistance	No replicons detected	No AMR genes detected	No AMR genes detected
Kp14	Muranga	Urine	Piperacillin, cefuroxime, cefuroxime axetil, ceftriaxone, cefepime, aztreonam, meropenem, trimethoprim, cefixime, levofloxacin	IncFIB(K)_1_Kpn3, IncN_1, IncFII_1_pKP91, IncFIB(pQil)_1_pQil,	*AAC(3)-IIe, AAC(6')-Ib-cr, CRP, CTX-M-15, FosA6, H-NS, OXA-1, QnrS1, TEM-1, aadA2, acrB, acrD, cpxA, dfrA12, emrR, mdtB, msbA, ramA, sul1, sul2*	Aminocoumarin, Aminoglycoside, Beta-lactams, Macrolide, Diaminopyrimidine, Fluoroquinolone, Fosfomycin, Macrolide, Nitroimidazole, Phenicol, Rifamycin, Sulfonamide, Tetracycline, Tobramycin, Triclosan,
Kp15	Kiambu	Urine	Amoxicillin/clavulanic, ampicillin/sulbactam, cefazolin, cefuroxime, cefuroxime axetil, cefoxitin, cefotaxime, ceftazidime, ceftriaxone, cefepime, aztreonam,	IncFIA(HI1)_1_HI1 (2), IncFII_1, IncR_1, Col440I_1,	*AAC(6')-Ib-cr, ANT(3'')-IIa, APH(3'')-Ib, APH(6)-Id, EreA2, ErmB, FosA6, OXA-1, OXA-232, QnrB17, TEM-1, aadA1, aadA2, aph(3'')-Ib, aph(6)-Id, armA, arr-2, blaCTX-M-15, blaNDM-5,*	Aminoglycoside, Macrolide, Beta-lactam, Phenicol, Diaminopyrimidine, Fluoroquinolone, Fosfomycin,

			meropenem, amikacin, gentamicin, ciprofloxacin, nitrofurantoin, piperacillin/ tazobactam, trimethoprim/sulfamethoxazole	ColKP3_1, Col(MG828)_1	*blaOXA-1, blaTEM-1, ble-MBL, cmlA5, dfrA12, ere(A), erm(B), mphE, mphA, msrE, oqxA10, oqxB19, qnrB1, sul1, sul2*	Glycopeptide, Lincosamide, Nitrofuran, Oxazolidinone, Peptide, Quinupristin, Rifamycin, Streptogramin, Sulfonamide, Tetracycline
Kp16	Kiambu	Urine	cefuroxime, cefotaxime, ceftazidime, ceftriaxone, cefepime, aztreonam, trimethoprim/sulfamethoxazole	IncFII_1_pKP91, IncFIB(K)_1_Kpn3	*AAC(6')-Ib-cr, APH(3'')-Ib, APH(3')-Ia, APH(6)-Id, CRP, CTX-M-15, OXA-1, QnrB17, TEM-1, acrB, baeR, cpxA, dfrA14, mdtB, mdtC, sul2*	Beta-lactams, Diaminopyrimidine, Aminocoumarin, Aminoglycoside, Cephalosporin, Fluoroquinolone, Glycylcycline, Macrolide, Phenicol, Rifamycin, Sulfonamide, Tetracycline, Triclosan
Kp17	Kiambu	Pus	ampicillin/sulbactam, cefazolin, cefuroxime, cefuroxime axetil, cefotaxime, ceftazidime, ceftriaxone, cefepime, aztreonam, gentamicin	ColRNAI_1, Col440I_1	*APH(3'')-Ib, APH(6)-Id, sul2*	Aminoglycoside, Sulfonamide

Kp18	Kisumu	Urine	Data not available	ColRNAI_1 (2), IncFIB(K)_1_Kpn3	*AAC(3)-IIe, AAC(6')-Ib-cr, APH(3'')-Ib, APH(6)-Id, CTX-M-15, H-NS, OXA-1, QnrS1, SHV-110, TEM-1, aac(6')-Ib-D181Y, aadA2, aph(3'')-Ib, aph(6)-Id, blaCTX-M-15, blaOXA-1, blaSHV-110, blaTEM-1, catA2, dfrA12, mph(A), mphA, qnrS1, sul1, sul2, tet(A)*	Aminoglycoside, Aztreonam, Beta-lactam, Diaminopyrimidine, Fluoroquinolone, Macrolide, Phenicol, Quinolone, Sulfonamide, Tetracycline
Kp19	Kiambu	Urine	amoxicillin/clavulanic, ampicillin/ sulbactam, cefazolin, cefuroxime, cefuroxime axetil, cefotaxime	IncFIB(K)_1_Kpn3, Col440I_1	*AAC(3)-IIe, APH(3'')-Ib, APH(6)-Id, CTX-M-15, TEM-1, acrB, dfrA14, sul2, tet(D)*	Aminoglycoside, Beta-lactams, Diaminopyrimidine, Fluoroquinolone, Phenicol, Rifamycin, Sulfonamide, Tetracycline, Triclosan
Kp20	Kiambu	Urine	cefuroxime, cefotaxime, ceftazidime, ceftriaxone, cefepime, aztreonam, ciprofloxacin, piperacillin/ tazobactam, trimethoprim/sulfamethoxazole	IncX3_1, IncFIB(K)_1_Kpn3	*AAC(6')-Ib-cr, OXA-1, QnrS1, aadA2, dfrA12, sul1*	Aminoglycoside, Beta-lactams, Diaminopyrimidine, Fluoroquinolone, Sulfonamide
Kp21	Trans-Nzoia	Pus	trimethoprim/sulfamethoxazole	IncR_1, IncFIA(HI1)_1_HI1	*AAC(6')-Ib-cr, APH(3'')-Ib, APH(6)-Id, CRP, CTX-M-15, CTX-M-88, FosA6, H-NS, QnrB17, SHV-187, TEM-1, aac(6')-Ib-D181Y, aadA16, acrB,*	Amikacin, Aminocoumarin, Aminoglycoside, Beta-lactam, Diaminopyrimidine,

					aph(3'')-Ib, aph(6)-Id, arr-3, baeR, blaCTX-M-216, blaTEM-1, cpxA, dfrA27, emrR, mdtB, mdtC, msbA, oqxA, oqxB, qnrB6, ramA, sul1, sul2	Fluoroquinolone, Fosfomycin, Macrolide, Nitrofuran, Nitroimidazole, Phenicol, Piperacillin, Rifamycin, Sulfonamide, Tetracycline, Triclosan
